# Factors associated with delayed neurologic improvement after complete endovascular reperfusion in anterior and posterior ischemic stroke

**DOI:** 10.3389/fneur.2025.1543743

**Published:** 2025-05-30

**Authors:** Sangil Park, Boseong Kwon, Jun Young Chang, Yunsun Song, Deok Hee Lee, Sang Hee Ha, Bum Joon Kim

**Affiliations:** ^1^Department of Neurology, Uijeongbu Eulji Medical Center, Eulji University School of Medicine, Uijeongbu, Republic of Korea; ^2^Department of Radiology, Asan Medical Center, College of Medicine, University of Ulsan, Seoul, Republic of Korea; ^3^Department of Neurology, Asan Medical Center, College of Medicine, University of Ulsan, Seoul, Republic of Korea; ^4^Department of Neurology, Gil Medical Center, Gachon University, Incheon, Republic of Korea

**Keywords:** endovascular thrombectomy, early neurological improvement, delayed neurological improvement, anterior circulation stroke, posterior circulation stroke

## Abstract

**Background:**

Mechanisms underlying delayed neurological improvement (DNI) after endovascular thrombectomy (EVT) in patients with anterior (ACS) and posterior circulation stroke (PCS) may differ. This study aims to compare the factors associated with DNI in patients with ACS and PCS.

**Materials and methods:**

Data of patients who underwent EVT with National Institute of Health Stroke Scale (NIHSS) score more than 6 and achieved successful reperfusion were retrospectively analyzed. DNI was defined as achieving favorable functional outcomes at 90 days, even without early neurological improvement. The factors associated with DNI in patients with ACS and PCS were investigated.

**Results:**

A total of 241 patients were included. The proportions of DNI (30.8% vs. 20.0%; *p* = 0.184) were not significantly different between patients with ACS and PCS. In patients with ACS, absence of atrial fibrillation (aOR = 0.500; 95% CI, 0.264–0.945; *p* = 0.033), statin use (aOR = 2.842; 95% CI, 1.174–6.882; *p* = 0.021), lower NIHSS score after 24 h (aOR = 0.816; 95% CI 0.757–0.880; *p* < 0.001), and shorter onset-to-door time (aOR = 0.999; 95% CI, 0.998–1.000; *p* = 0.025) were significantly associated with DNI. In patients with PCS, male sex (aOR = 31.809; 95% CI, 1.816–557.074; *p* = 0.018) and lower initial NIHSS scores (aOR = 0.626; 95% CI, 0.410–0.957; *p* = 0.031) were significantly associated with DNI.

**Conclusion:**

The proportions of DNI were similar in patients with ACS and PCS. However, the factors associated with DNI were different between the two groups.

## Introduction

Endovascular thrombectomy (EVT) has become an established treatment for acute ischemic stroke with emergent large vessel occlusion (1). When complete arterial recanalization is achieved, it is often accompanied with early neurological improvement (ENI), a robust predictor of favorable long-term clinical outcomes (2). Nevertheless, cases have been noted where patients do not exhibit a clinical response within the initial hours following successful recanalization (3).

Lack of an early clinical response after recanalization does not always indicate a poor long-term outcome. Instead, it can be attributed to delayed neurological improvement (DNI). This phenomenon is associated with the “stunned brain” phenomenon, which involves various factors such as the resolution of brain edema, delayed improvement in microcirculation within ischemic tissues, and neuronal reorganization (4). The DNI occurring after EVT were associated with long-term outcomes, although most previous studies were focused on the patients with anterior circulation stroke (ACS) (2, 5). Moreover, it remains unclear whether the factors related to the outcome for DNI differ in patients receiving EVT with posterior circulation stroke (PCS).

This study aimed to compare clinical and radiographic findings and clinical outcomes after complete endovascular reperfusion. We also aimed to identify the factors associated with DNI in patients with ACS and in those with PCS.

## Materials and methods

### Patients and clinical data

We reviewed patients with acute ischemic stroke who were admitted to single-center, Seoul, Korea between January 2017 and June 2022. Patients were included in this study if they were ≥18 years-old; presented a National Institutes of Health Stroke Scale (NIHSS) score of >6 at baseline; underwent EVT with an arterial occlusion in one of the following locations: internal carotid artery (cervical or intracranial), middle cerebral artery (M1 or proximal M2 segment), basilar artery, and vertebral artery, and achieved modified thrombolysis in cerebral infarction 2b/3 reperfusion (6). All EVT procedures were conducted in accordance with national clinical practice guidelines and local protocols (7). The local ethics committee approved this study (IRB number: 2022-0236), and informed consent was waived owing to the retrospective nature of the study.

Demographics and risk factors were documented from medical records. We studied NIHSS scores at admission, 24 h after EVT, and at discharge (8). The presumed cause of stroke was categorized according to the Trial of Org 10172 in the Acute Stroke Treatment (TOAST) classification (9). Processing times (from stroke onset to hospital arrival, from door to groin puncture, stroke onset to groin puncture, and puncture to reperfusion times) were also assessed.

### Imaging variables

Multi-modal magnetic resonance imaging (MRI; Philips Healthcare, Eindhoven, The Netherlands) was performed, which included DWI, perfusion-weighted (PWI), FLAIR, gradient-echo imaging, and time-of-flight MRA (intracranial) and contrast-enhanced (extracranial) MRA (10). We used the Olea Sphere^®^ imaging system (Olea Medical SAS, La Ciotat, France) for automatic post processing of PWI and DWI studies (11). To measure the DWI lesion volume, we set a value threshold of the apparent diffusion coefficient value. The volume of hypoperfused brain tissue was calculated using the T max (defined as a >6-s delay) (12).

### Revascularization procedure and outcome

To perform digital subtraction angiography, we used a 6F ENVOY guiding catheter (Cordis, Miami Lakes, FL, United States) or an 8F Merci balloon guide catheter (Concentric Medical, Mountain View, CA, United States). In certain cases, other rescue strategies (angioplasty, stent insertion, and glycoprotein IIb/IIIa receptor inhibitor treatment) were used when the mechanical thrombectomy failed or if residual stenosis prevented adequate reperfusion. Reperfusion status was assessed in final cerebral angiography and graded according to the modified Thrombolysis in Cerebral Infarction (mTICI) scale (13). Successful reperfusion was defined as a scale score of 2b or 3 (13). An MRI follow-up was conducted to validate the presence of any intracranial hemorrhage subsequent to EVT, and the classification of hemorrhage was determined according to the definition outlined in the European Cooperative Acute Stroke Study (14).

ENI was defined a reduction of at least 8 NIHSS points or NIHSS equals to 0–1 at 24 h after EVT (15–17). DNI was defined if, despite absence of ENI during the first 24 h, patients achieved favorable functional outcomes (mRS 0–2) at 90 days (5). Functional outcome was measured by a stroke neurologist, either during patients’ physical visits to our stroke prevention clinic or by telephone with a structured interview.

### Statistical analysis

The characteristics of patients with and without DNI were compared. The chi-square test (Fisher’s exact test) and the Student’s *t*-test was used adequately. In this study, we performed univariable and multivariable analyses to evaluate the factors associated with DNI in each group. The selection of adjusted variables for multivariable analysis was based on the results of univariable analysis and demographic factors, where only variables with a *p*-value <0.1, age, and sex were included in the final model. We assessed collinearity and excluded variables with a tolerance <1, variance inflation factor (VIF) ≥10, or a correlation coefficient ≥0.9. We used the IBM SPSS Statistics software, version 21.0 (IBM, Armonk, NY), and significance was set at *p* < 0.05 (18).

## Results

**Figure 1:**
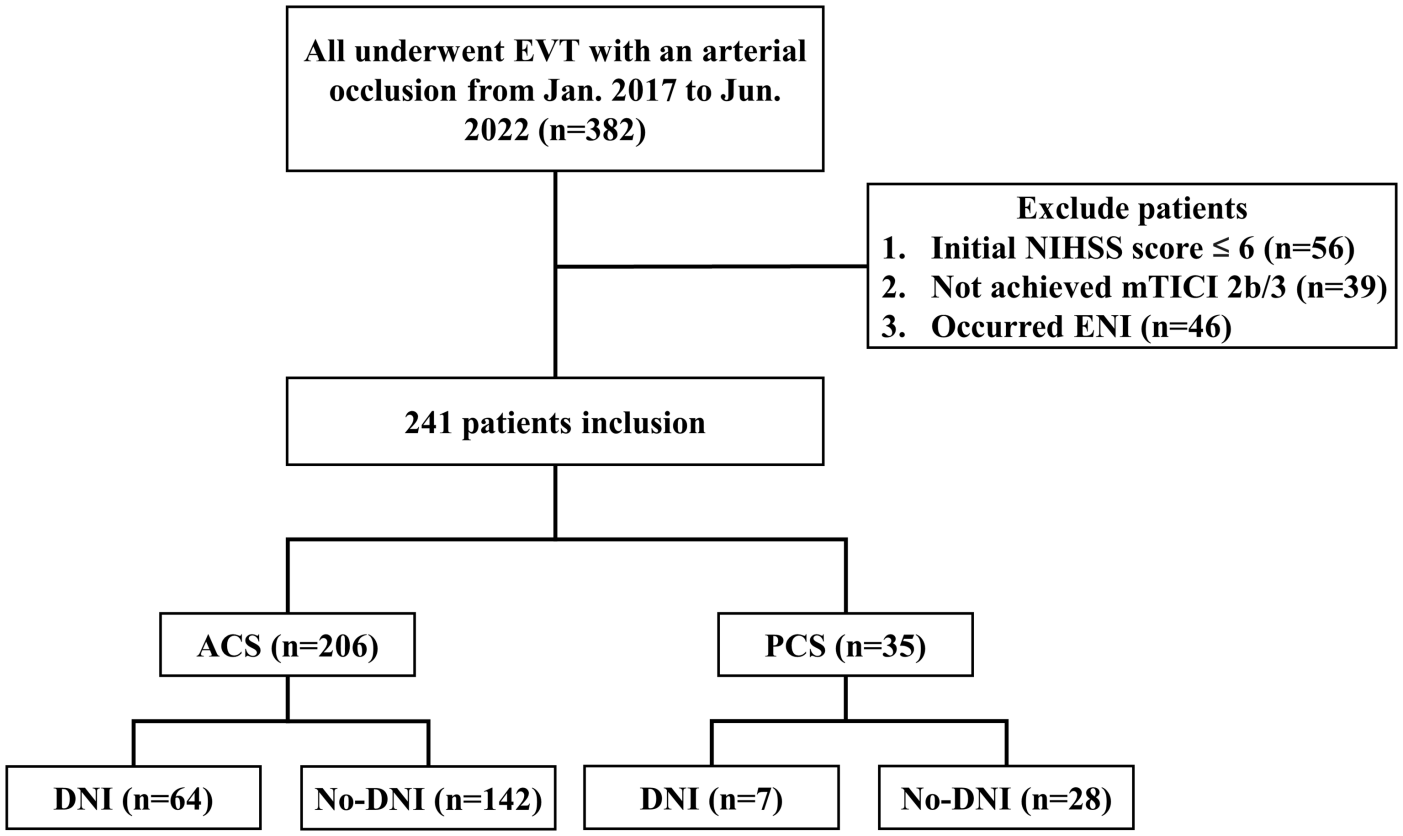
Study flowchart. DNI, delayed neurological improvement; ENI, early neurological improvement; EVT, endovascular thrombectomy, mTICI, modified treatment in cerebral infarction; NIHSS, National Institutes of Health Stroke Scale.

A total of 382 patients underwent EVT during the study period, with 56 (14.7%) exhibiting an NIHSS score of < 6 points, 39 (10.2%) displaying a mTICI scale below 2b and 46 (12.0%) experienced ENI. Ultimately, the study included 241 (63.1%) patients (Figure 1).

The mean age of the patients was 70 ± 4 years, and 146 (60.6%) were male. The median NIHSS score at admission was 12.0 points (interquartile range, 8.0–16.0). Among them, 206 (85.5%) patients had ACS, and 35 (14.5%) patients had PCS. DNI was observed in 71 (29.5%) patients; 64 (30.8%) in ACS and 7 (20.0%) in PCS patients.

Among the seven PCS patients with DNI, the lesions were located in various areas, including the cerebellum (*n* = 6), dorsolateral pons (*n* = 2), temporo-occipital lobe (*n* = 2), and medial thalamus (*n* = 2).

### Comparison between patients with and without DNI

No significant differences in demographics and vascular risk factors were observed between the patients with and without DNI. Compared to patients without, those with DNI had more common prior statin medications (8.2 vs. 21.1%; *p* = 0.005), lower proportion of large artery atherosclerosis (30.6 vs. 15.5%) and higher rate of other determined etiology (10.0 vs. 21.1%; *p* = 0.027), shorter door to groin puncture time (230 ± 278 vs. 164 ± 171 min; *p* = 0.026), and onset to recanalization time (422 ± 286 vs. 341 ± 228 min; *p* = 0.036). There was no significant difference in initial NIHSS score before EVT. However, the NIHSS score after 24 h [13 (9–18) vs. 7 (3–11); *p* < 0.001] and at discharge [10 (5–14) vs. 4 (1–10); *p* < 0.001] were significantly lower in those with DNI than in those without DNI (Table 1).

**Table 1:** Clinical characteristics of patients with and without DNI.

Variable	DNI^–^ (*N* = 170)	DNI^+^ (*N* = 71)	*p*-value
Age (years)	70 ± 11	70 ± 12	0.737
Sex (male)	106 (62.4)	40 (56.3)	0.384
Hypertension	106 (62.4)	49 (69.0)	0.325
Diabetes mellitus	48 (28.2)	24 (33.8)	0.389
Hyperlipidemia	36 (21.2)	18 (25.4)	0.479
Atrial fibrillation	83 (49.1)	30 (42.3)	0.331
Smoking	61 (35.9)	25 (35.2)	0.921
Previous stroke	47 (27.6)	16 (22.5)	0.410
Previous medication history			
Antiplatelet (mono)	26 (15.3)	15 (21.1)	0.394
Antiplatelet (dual)	39 (22.9)	12 (16.9)	
Anticoagulation	80 (47.1)	37 (52.1)	0.474
Statin	14 (8.2)	15 (21.1)	**0.005**
Initial NIHSS score, median (IQR)	13 (9–16)	12 (7–16)	0.088
Intravenous tPA	34 (20.0)	19 (26.8)	0.248
TOAST classification			**0.027**
Large artery atherosclerosis	52 (30.6)	11 (15.5)	
Cardioembolism	91 (53.5)	41 (57.7)	
Other determined	17 (10.0)	15 (21.1)	
Undetermined	10 (5.9)	4 (5.6)	
Lesion location			0.184
Anterior circulation	142 (83.5)	64 (90.1)	
Posterior circulation	28 (16.5)	7 (9.9)	
DWI lesion volume	23.89 ± 32.28	19.50 ± 26.23	0.352
PWI lesion volume	114.60 ± 87.80	104.17 ± 79.25	0.429
Endovascular treatment			
Thrombectomy	152 (89.4)	64 (90.1)	0.094
Angioplasty/stenting	37 (21.8)	12 (16.9)	0.392
Additional chemical lysis	8 (4.7)	6 (8.5)	0.257
Time interval, minute			
Onset to door	437 ± 389	333 ± 336	0.050
Door to groin puncture	230 ± 278	164 ± 171	**0.026**
Puncture to recanalization	67 ± 57	58 ± 39	0.201
Onset to recanalization	422 ± 286	341 ± 228	**0.036**
NIHSS after 24 h, median (IQR)	13 (9–18)	7 (3–11)	**<0.001**
Discharge NIHSS, median (IQR)	10 (5–14)	4 (1–10)	**<0.001**
First pass effect	52 (30.8)	30 (42.3)	0.087
Any hemorrhage^a^	41 (24.1)	12 (16.9)	0.218
Symptomatic intracerebral hemorrhage^a^	6 (3.5)	0 (0.0)	0.109

Results are presented as numbers and percentages or as means ± standard deviations or medians (interquartile ranges). DWI, diffusion-weighted imaging; IQR, interquartile range; NIHSS, National Institutes of Health Stroke Scale; PWI, perfusion-weighted imaging; TOAST, Trial of Org 10172 in Acute Stroke Treatment; tPA, tissue plasminogen activator.

a Any hemorrhage was scored according to the definition provided by the European Cooperative Acute Stroke Study as follows: small petechial hemorrhagic infarction, confluent petechial hemorrhagic infarction, small parenchymal hemorrhage (<30% of infarct, mild mass effect), and large parenchymal hemorrhage (>30% of infarct, marked mass effect). Clinical deterioration or adverse events indicating clinical worsening (e.g., drowsiness, increase of hemiparesis) or an increase in NIHSS score of ≥4 points were defined as symptomatic intracerebral hemorrhage. The values with bold type represent statistically significant results with a *p*-value < 0.05.

### Comparison of patient characteristics in ACS vs. PCS

In comparison to patients with PCS, those with ACS showed lower initial NIHSS scores [12 (8–16) vs. 13 (8–19); *p* < 0.001], higher DWI (24.71 ± 32.45 vs. 10.85 ± 11.57 cc; *p* < 0.001) and PWI lesion volume (121.19 ± 88.71 vs. 60.93 ± 33.27 cc; *p* < 0.001), higher thrombectomy rate (91.3 vs. 80.0%; *p* = 0.043), shorter onset to recanalization time (379 ± 255 vs. 506 ± 340 min; *p* = 0.041), lower NIHSS scores 24 h after EVT [11 (7–16) vs. 16 (7–23); *p* = 0.011] and at discharge [8 (4–12) vs. 13 (6–22); *p* = 0.005; Table 2].

**Table 2:** Clinical characteristic of patients in anterior and posterior circulation.

Variable	Anterior *n* = 206	Posterior *n* = 35	*p*
Age (years)	70 ± 11	68 ± 13	0.343
Sex (male)	126 (61.2)	20 (57.1)	0.653
Hypertension	134 (65.0)	21 (60.0)	0.564
Diabetes mellitus	61 (29.6)	11 (31.4)	0.828
Hyperlipidemia	44 (21.4)	10 (28.6)	0.344
Atrial fibrillation	98 (47.8)	15 (42.9)	0.588
Smoking	75 (36.4)	11 (31.4)	0.570
Previous stroke	51 (24.8)	12 (19.0)	0.236
Previous medication history			
Antiplatelet (mono)	35 (17.0)	6 (17.1)	0.983
Antiplatelet (dual)	44 (21.4)	7 (20.0)	
Anticoagulation	104 (50.5)	13 (37.1)	0.144
Statin	24 (11.7)	5 (14.3)	0.658
Initial NIHSS score, median (IQR)	12 (8–16)	13 (8–19)	**<0.001**
Intravenous tPA	43 (20.9)	10 (28.6)	0.309
TOAST classification			0.227
Large artery atherosclerosis	49 (23.8)	14 (40.0)	
Cardioembolism	116 (56.3)	16 (45.7)	
Other determined	28 (13.6)	4 (11.4)	
Undetermined	13 (6.3)	1 (2.9)	
DWI lesion volume	24.71 ± 32.45	10.85 ± 11.57	**<0.001**
PWI lesion volume	121.19 ± 88.71	60.93 ± 33.27	**<0.001**
Clinical-diffusion mismatch^a^	91 (54.8)	20 (64.5)	0.318
Endovascular treatment			
Thrombectomy	188 (91.3)	28 (80.0)	**0.043**
Angioplasty/Stenting	40 (19.4)	4 (25.7)	0.392
Additional chemical lysis	10 (4.9)	4 (11.4)	0.124
Time interval, minute			
Onset to door	402 ± 381	432 ± 353	0.662
Door to groin puncture	204 ± 251	250 ± 265	0.320
Puncture to recanalization	64 ± 54	68 ± 47	0.646
Onset to recanalization	379 ± 255	506 ± 340	**0.041**
Delayed neurological improvement	64 (30.8)	7 (20.0)	0.184
NIHSS after 24 h, median (IQR)	11 (7–16)	16 (7–23)	**0.011**
Discharge NIHSS, median (IQR)	8 (4–12)	13 (6–22)	**0.005**
First pass effect	68 (33.2)	14 (40.0)	0.431
Any hemorrhage^b^	42 (20.4)	11 (21.4)	0.145
Symptomatic intracerebral hemorrhage^b^	4 (1.9)	2 (5.7)	0.185

Results are presented as numbers and percentages or as means ± standard deviations or median (interquartile ranges). DWI indicates diffusion-weighted imaging; IQR, interquartile range; NIHSS, National Institutes of Health Stroke Scale; PWI, perfusion-weighted imaging; TOAST, Trial of Org 10172 in Acute Stroke Treatment; tPA, tissue plasminogen activator.

a NIHSS ≥8 combined with a DWI volume ≤25 mL was defined as a clinical-diffusion mismatch. ^b^Any hemorrhage was scored according to the definition provided by the European Cooperative Acute Stroke Study as follows: small petechial hemorrhagic infarction, confluent petechial hemorrhagic infarction, small parenchymal hemorrhage (<30% of infarct, mild mass effect), and large parenchymal hemorrhage (>30% of infarct, marked mass effect). Clinical deterioration or adverse events indicating clinical worsening (e.g., drowsiness, increase of hemiparesis) or an increase in NIHSS score of ≥4 points were defined as symptomatic intracerebral hemorrhage. The values with bold type represent statistically significant results with a *p*-value < 0.05.

### Factors associated with DNI

In patients with ACS, the prior use of statins, other determined etiology, and lower NIHSS score after 24 h were associated with DNI. In the multivariable logistic regression analysis, prior use of statins [adjusted odds ratio (aOR) = 2.842; 95% confidential interval (CI), 1.174–6.882; *p* = 0.021], lower NIHSS after 24 h (aOR = 0.816; 95% CI 0.757–0.880; *p* < 0.001), and a shorter onset-to-door time (aOR = 0.999; 95% CI, 0.998–1.000; *p* = 0.025) were significantly associated with DNI (Table 3).

**Table 3:** Factors associated with DNI in anterior circulation and posterior circulation stroke.

Factors	Anterior circulation				Posterior circulation			
	Univariable analysis		Multivariable analysis^a^		Univariable analysis		Multivariable analysis^b^	
	OR (95% CI)	*p*	OR (95% CI)	*p*	OR (95% CI)	*p*	OR (95% CI)	*p*
Age (years)	0.996 (0.970–1.022)	0.740			0.990 (0.927–1.056)	0.756	—	
Sex (male)	1.014 (0.554–1.857)	0.964			12.667 (1.321–121.469)	0.028	**31.809 (1.816–557.074)**	**0.018**
Hypertension	1.568 (0.826–2.979)	0.169			0.417 (0.077–2.246)	0.308		
Diabetes mellitus	1.535 (0.816–2.887)	0.183			0.300 (0.032–2.857)	0.295		
Hyperlipidemia	1.357 (0.673–2.735)	0.393			1.000 (0.160–6.255)	>0.999		
Atrial fibrillation	0.597 (0.327–1.089)	0.093	**0.500 (0.264–0.945)**	**0.033**	4.500 (0.734–27.577)	0.104		
Smoking history	1.179 (0.642–2.168)	0.595			NA	>0.999		
Previous stroke history	0.795 (0.394–1.602)	0.520			0.720 (0.117–4.412)	0.722		
Previous medication history								
Antiplatelet (mono)	1.504 (0.694–3.263)	0.301			0.680 (0.064–7.254)	0.749		
Antiplatelet (dual)	0.752 (0.345–1.640)	0.474			0.567 (0.055–5.883)	0.634		
Anticoagulation	0.972 (0.539–1.754)	0.925			6.250 (0.999–39.094)	0.050		
Statin	3.036 (1.277–7.214)	0.012	**2.842 (1.174–6.882)**	**0.021**	3.333 (0.438–25.394)	0.245		
Initial NIHSS score	0.986 (0.923–1.054)	0.681			0.728 (0.531–0.998)	0.048	**0.626 (0.410–0.957)**	**0.031**
Intravenous tPA	1.614 (0.802–3.246)	0.180			1.000 (0.160–6.255)	>0.999		
TOAST classification								
Large artery atherosclerosis	reference				reference			
Cardioembolism	1.755 (0.790–3.899)	0.167			5.909 (0.597–58.484)	0.129		
Other determined	3.900 (1.413–10.768)	0.009			4.333 (0.207–90.847)	0.345		
Undetermined	1.733 (0.442–6.804)	0.430			NA	>0.999		
DWI lesion volume	0.995 (0.984–1.006)	0.376			0.884 (0.754–1.036)	0.128		
PWI lesion volume	0.998 (0.994–1.002)	0.348			0.979 (0.951–1.008)	0.150		
Clinical-diffusion mismatch^c^	0.750 (0.391–1.439)	0.387			0.309 (0.054–1.753)	0.185		
Endovascular treatment								
Thrombectomy	0.892 (0.319–2.494)	0.828			1.636 (0.164–16.345)	0.675		
Angioplasty/Stenting	0.809 (0.376–1.741)	0.587			0.417 (0.043–4.034)	0.450		
Additional chemical lysis	2.322 (0.648–8.323)	0.196			1.389 (0.122–15.812)	0.791		
Time interval, hours (range)								
Onset to door	0.999 (0.998–1.000)	0.051	**0.999 (0.998–1.000)**	**0.025**	1.000 (0.997–1.002)	0.880		
Door to groin puncture	0.999 (0.997–1.000)	0.153			0.994 (0.980–1.007)	0.357		
Puncture to recanalization	0.996 (0.989–1.004)	0.353			0.988 (0.968–1.009)	0.265		
Onset to recanalization	0.999 (0.998–1.000)	0.118			0.998 (0.994–1.002)	0.265		
NIHSS after 24 h	0.859 (0.808–0.913)	<0.001	**0.816 (0.757–0.880)**	**<0.001**	0.485 (0.230–1.024)	0.058		
FPE	1.613 (0.871–2.985)	0.128			2.400 (0.445–12.939)	0.308		
Any hemorrhage	0.743 (0.347–1.592)	0.445			0.300 (0.032–2.857)	0.295		
Symptomatic intracerebral hemorrhage	NA	>0.999			NA	>0.999		
Death within 3 months	0.610 (0.192–1.930)	0.400			NA	>0.999		

Results are presented as odds ratio and 95% confidence intervals (CIs). NIHSS, National Institutes of Health Stroke Scale; DWI, diffusion-weighted image; PWI, perfusion-weighted image; NA, not available. The values with bold type represent statistically significant results with a *p*-value < 0.05.

a Multivariable logistic regression adjusted for age, sex, atrial fibrillation, statin use, onset to door time, and NIHSS after 24 h.

b Multivariable logistic regression adjusted for age, sex, anticoagulation use, and initial NIHSS score.

^c^NIHSS ≥8 combined with a DWI volume ≤25 mL was defined as a clinical-diffusion mismatch.

Otherwise, in patients with PCS, being male and having a lower initial NIHSS score were associated with DNI. The multivariable logistic regression analyses revealed that being male (aOR = 31.809; 95% CI, 1.816–557.074; *p* = 0.018) and having a lower initial NIHSS score (aOR = 0.626; 95% CI, 0.410–0.957; *p* = 0.031) were significantly associated with DNI (Table 3).

## Discussion

In this study, 29.5% patients receiving EVT with achieving successful recanalization showed DNI. The incidence of DNI between ACS and PCS was similar, but the factors associated with DNI were different between ACS and PCS. For patients with ACS, the use of prior statins, shorter onset-to door time and lower NIHSS after 24 h were associated with DNI. On the other hand, being male and lower initial NIHSS score were associated with DNI in patients with PCS.

Even though the extent does not meet the definition of ENI, the improvement in NIHSS scores 24 h after EVT was associated with DNI, but only in ACS group. Early improvement of reperfusion in the ischemic penumbra, which can lead to the rapid resolution of cortical symptoms predominantly seen in ACS may explain the association between 24 h neurological improvement and DNI (19).

In contrast, in PCS, a lower initial NIHSS score was significantly associated with DNI. The location of the stroke, influencing the initial severity, may be a more critical factor in determining recovery in PCS patients. While the general course of clinical recovery is relatively rapid during the first few weeks and then decelerates 1–3 months later, severe paralysis has been identified as valid associated factors of poor functional outcomes (20). In our current study, seven PCS patients with DNI exhibited various lesion locations, including the cerebellum (*n* = 6), dorsolateral pons (*n* = 2), temporo-occipital lobe (*n* = 2), and medial thalamus (*n* = 2), with the corticospinal tracts preserved in these patients. This finding suggests that the preservation of motor pathways may play a role in facilitating early neurological recovery in PCS patients.

We also found that rapid reperfusion was the important factor for DNI in ACS. A previous study has indicated that patients with PCS had a significantly longer onset-to-door time than those with ACS, and despite prolonged symptom onset to recanalization initiation intervals in PCS cases, functional outcomes at 3 months were comparable between ACS and PCS groups (21, 22). A better collateral flow in PCS may result in a slower evolution of irreversible ischemia, contributing to better tolerance to the time window (23).

Several studies have suggested a potential beneficial effect of premorbid statin use on functional outcomes following acute stroke (24). These benefits may arise from the pleiotropic effects of statins, including their anti-inflammatory, antioxidant, and neuroprotective properties, which could mitigate the extent of neuronal damage and improve post-stroke recovery (25). We found that prior statin use was more associated with DNI in patients with ACS, but less in those with PCS. One plausible explanation for this contrast lies in the disparity of the extent of lesion volume between ACS and PCS. Although not statistically significant, we observed that patients with ACS exhibited larger ischemic core and penumbra compared to those with PCS, potentially resulting in more extensive neuronal damage. Hence, the pleiotropic effects of statins might exert a more pronounced impact in the context of ACS, characterized by greater neuronal injury and inflammation.

An intriguing observation was that male sex was significantly associated with long-term recovery compared to female sex if there was no improvement within the first 24 h following complete arterial recanalization. This aligns with prior research indicating worse outcomes for women after stroke in general, as well as after endovascular therapy (2, 26). Also, this disparity may be influenced by both biological and social factors. Biologically, sex hormones such as estrogen have been suggested to modulate neuroprotection and inflammatory responses after stroke, potentially influencing recovery trajectories. On the social side, differences in participation in post-stroke rehabilitation, caregiver support, and access to healthcare resources may further impact these outcomes (27). However, this variable was not specifically analyzed due to the retrospective nature of our study. Moreover, given the small number of patients with DNI, we cannot exclude the possibility that this association might be the result of an artifact or an unexamined selection bias. Consequently, these finding warrants confirmation in future studies on endovascular therapy.

This study has several limitations. First, the limited sample size and observational design at a single center may introduce selection bias and reduce statistical power, particularly in the PCS group (*n* = 35), limiting the generalizability of the findings. Second, multiple comparisons were not adjusted for, increasing the risk of type I errors, and some significant results may not survive correction. Third, the small number of DNI events (*n* = 7) in the PCS group raises concerns about the robustness of multivariable modeling, as indicated by wide confidence intervals and potential quasi-complete separation. Fourth, PCS patients frequently present with symptoms such as dizziness, diplopia, visual field defects, and gait ataxia, which are often underrepresented or underappreciated in NIHSS score assessment. This highlights the challenges associated with using NIHSS score to assess stroke severity and recovery in cases of PCS. Fifth, we identified factors associated with DNI; however, these findings need to be validated through randomized controlled trials to establish clear clinical practice. Finally, the definition of ENI and DNI varied among studies; but we chose to definitions based on prior research supporting its relevance (2, 28).

## Conclusion

We found that the proportions of DNI were similar in patients with ACS and PCS, but the factors associated with DNI may differ. For ACS patients, prior statin use, rapid reperfusion, and early response to neurological improvement may be crucial. In contrast, for PCS patients, initial stroke severity and male sex were significant factors.

## Data Availability

The data analyzed in this study is subject to the following licenses/restrictions: the dataset used in this study is derived from patient medical records and is subject to restrictions due to patient confidentiality, institutional policies, and ethical guidelines. Access to the data requires approval from the Institutional Review Board (IRB) of Asan Medical Center. Requests to access these datasets should be directed to BKi, medicj80@hanmail.net.
